# New Biomolecules and Drug Delivery Systems as Alternatives to Conventional Antibiotics

**DOI:** 10.3390/antibiotics11030318

**Published:** 2022-02-28

**Authors:** Helena P. Felgueiras

**Affiliations:** Centre for Textile Science and Technology (2C2T), Campus de Azurém, University of Minho, 4800-058 Guimaraes, Portugal; helena.felgueiras@2c2t.uminho.pt

New approaches to deal with the growing concern associated with antibiotic-resistant bacteria are in high demand. For many years, antibiotics have been the gold standard for treating infections. However, their excessive consumption and misuse have contributed to the rise of microorganisms resistant to antibiotic action, leading to a global health crisis. Engineering new drug delivery platforms and uncovering alternative biomolecules with antimicrobial and regenerative potentials is becoming extremely urgent. This Special Issue aims at expanding our understanding of the antimicrobial action of specialized biomolecules, recently engineered or chemically modified from their ancient origins, and to introduce a broad audience to new systems of drug delivery. 

In this collection of research, many important findings can be highlighted, namely the emergence of flavonoid-coated gold nanoparticles [1] and the concurrent effect of quercetin (also a bioflavonoid)- and magnesium-doped calcium silicates as highly effective antibacterial agents against Gram-negative bacteria [2], the engineering of silver nanoparticles via a new green synthesis methodology that improved the performance of these cues against multidrug-resistant bacteria [3], and the loading of poly-ε-caprolactone nanoparticles with 4-nerolidylcatechol that specifically inhibited the growth of *Microsporum canis* [4]. Another strategy against multidrug-resistant microbials was introduced by Han et al., whose findings demonstrated grapefruit seed extracts as effective antibacterial agents, even at low concentrations [5], while Lin et al. revealed the potentialities of aerosolized hypertonic saline against multidrug-resistant *Acinetobacter baumannii*, unveiling a new vehicle of delivery for conventional antibiotics capable of improving their effectiveness [6]. 

Discovering new pharmaceutical strategies to fight infection is a very challenging and time-consuming process. Considering DNA gyrase and topoisomerase IV are known targets for novel antibacterial drug design, Saleh et al. demonstrated the effectiveness of diphenylphosphonates as DNA gyrase inhibitors, with great potentially for new pharmaceutical formulations [7]. Furthermore, Baranova et al. followed a different route and resorted to live biosensors for ultra-high-throughput screening for deep profiling of antibacterial activity and antibiotic discovery [8].

Even though most of the focus of this Special Issue is on developing and unveiling new antimicrobial cues capable of mitigating or irradicating infection in humans, plants and animal products are also affected by similar issues, with pesticides and other chemical agents becoming highly ineffective against microbial plagues. Assessments of pathogenic resistance have been made by Lianou et al. on bulk-tank milk of goat herds, to identify potentially dangerous factors affecting the product quality [9]. These assessments are essential to better understand the mechanisms of action of microbial species, and hence, propose personalized solutions. Wang et al. investigated a novel polyene agriculture antibiotic, tetramycin, and determined that it could prevent several diseases in kiwi plants [10]. Allicin and chitosan were also found to increase the resistance of *Rosa roxburghii* against powdery mildew, being highlighted as a potential green, cost-effective and environmentally friendly strategy to raise production yields [11]. 

Finally, recent advances in antibiotic- and heavy-metal-loaded titanium surfaces were examined as potential mechanisms for preventing local infection in bone-related applications [12], while for tissue engineering and wound healing, fiber–hydrogel composites were identified as prospective effective solutions [13]. Even though this Special Issue has provided significant evidence of the high level of research and dedication in finding potential options and solutions to the present antibiotic crisis, introducing readers to both techniques and molecules with an active profile against microbial agents, we anticipate that there are many antimicrobial cues and delivery systems with precise targets and mechanisms of action still to uncover. 
